# Adverse events and tolerability of ketogenic diets – a systematic literature analysis

**DOI:** 10.1186/s40795-026-01277-5

**Published:** 2026-02-20

**Authors:** Carolin Schopf, Marc Assmann, Nadja Wolke, Marius Frenser, Thorsten Marquardt, Tobias Fischer

**Affiliations:** 1 Center for Nutrition and Therapy (NuT), University of Applied Sciences Muenster, Corrensstraße 25, Muenster, 48149 Germany; 2 Department of Pediatrics, University Hospital Muenster, Albert-Schweitzer-Campus 1, Muenster, 48149 Germany

**Keywords:** Ketogenic diet, Adverse events, Adverse effects, Tolerance, Database analysis, Literature analysis

## Abstract

**Background:**

Ketogenic diets (KDs) are becoming increasingly popular in the treatment of various diseases, not just pharmacoresistant epilepsy. Despite the growing use of this dietary approach and its economic rationale, a systematic overview of the associated adverse events remains conspicuously absent.

**Methods:**

A systematic literature search was conducted in accordance with PRISMA guidelines in the electronic database PubMed. Prospective interventional studies documenting adverse events associated with ketogenic diets published between 2019 and 2024 were included. The recorded adverse events were categorized according to CTCAE criteria and analyzed based on frequency and diversity by diet type.

**Results:**

A total of 36 studies involving 42 ketogenic interventions (classic KD, modified Atkins diet, MCT-based KD, LGIT) were evaluated. The included studies covered a wide range of age groups and conditions. At least one adverse event was documented in 43% of the participants (0–89%, 0.91 ± 0.71 adverse events per person). The most prevalent adverse events were gastrointestinal (40%), followed by neurological (17%), and metabolic and nutritional (12%) disorders. Constipation (24%) was the most commonly reported individual adverse event. A higher rate and severity of adverse events was observed in patients following stricter diets. The tolerability of the intervention was influenced by several factors, including age, the presence of underlying diseases, and the duration of the intervention.

**Conclusions:**

The analysis indicates that adverse events frequently occur alongside KD and encompass a broad spectrum, with gastrointestinal complaints predominating. However, the significance of the findings is limited by the heterogeneity of dietary regimens, the lack of standardization, and the occasional inadequate documentation of adverse events. In clinical practice, it is recommended that adverse events are recorded systematically and monitored regularly.

## Introduction

 The ketogenic diet (KD) is a very high-fat and very low-carbohydrate diet that originated as a therapeutic treatment for epilepsy [1, 2]. As early as 500 BC, it was observed that fasting could reduce epileptic seizures. However, it was not until the 20th century that researchers recognized that a fasting-like state could also be achieved through a KD [3, 4].

Biochemically, a sharp reduction in carbohydrate intake causes blood glucose and insulin levels to drop. This stimulates increased fatty acid oxidation and increased production of ketone bodies (β-hydroxybutyrate (BHB), acetoacetate (AcAc), and acetone) in the liver [5]. While BHB and AcAc serve as alternative energy sources, especially for the brain and muscles, acetone is produced as a spontaneous by-product [6]. The physiological ketosis range is approximately 0.5–8 mmol/L BHB [7].

In 1921, Dr. Wilder first published the KD and its corresponding terminology for the treatment of childhood epilepsy [3, 8]. Numerous research projects followed in the years that ensued, but interest in the KD waned in the 1950s with the development of effective antiepileptic drugs [4]. The KD then once again became the focus of nutritional therapy as a treatment option for children with drug-resistant epilepsy in the 1990s [3, 4].

Despite their long history of use, there is no uniform definition of KDs or their associated macronutrient distributions [9]. In essence, any diet that induces a ketogenic metabolic state can be categorised as ketogenic [4]. While individuals may respond differently to this type of diet, most studies on KDs limit carbohydrate intake to less than 10% of daily energy intake (E%) in order to achieve a fasting-like state. Depending on the person’s body weight, this corresponds to approximately 50 g of carbohydrates per day [9–11]. In the context of nutritional therapy, the four main types of KDs are the classic KD (CKD), the modified Atkins diet (MAD), the medium-chain triglyceride diet (MCT-KD), and the low glycemic index treatment (LGIT) [12]. In addition to clinical applications, KDs are now believed to have numerous health benefits and prevent disease [1]. For instance, an analysis of 290 studies on KDs, covering various research areas such as obesity, epilepsy, sports, type 2 diabetes, and cancer, was conducted between 2019 and 2024 [13]. While many claims have not yet been sufficiently investigated, justified interest in the KD is growing, as is research interest [10].

The KD is associated with adverse events (AEs). However, serious complications are considered rare [8, 14]. During the initial phase of adapting to the diet, symptoms such as headaches, irritability, dizziness, brain fog, sleep disturbances, lack of energy, and fatigue may occur [9, 10, 15]. These symptoms are referred to as “keto flu” in a non-clinical setting [10, 15]. Additionally, lethargy, hyperuricemia, halitosis, dehydration, hypoglycemia, and gastrointestinal symptoms such as nausea, diarrhea, vomiting, constipation, and abdominal pain are often reported during this stage [8, 14, 16]. In addition to a shift in fluid and electrolyte balance [17], some of the symptoms in children may be attributed to a refusal to eat and drink [14]. In the long term, the following have been observed: increased infections, constipation, hypercholesterolemia, hypertriglyceridemia, cardiomyopathy, atherosclerotic changes, growth retardation in children, changes in platelet function, optic neuropathy, osteopenia, disorders of neutrophil granulocyte function, pancreatitis, secondary carnitine deficiency, hypoproteinemia, hypomagnesemia, kidney stones, gallstones, hair loss, and hepatitis [8, 10, 14, 16]. These AEs are suspected to be related to high fat intake, limited food choices, and the immunomodulatory effect of KD [10]. Currently, there is no consistent data on the frequency of these AEs. For this reason, they are not specified in the guidelines of national neurological societies (as of August 2025) [14, 16].

Due to the scarcity of cross-indication AE syntheses, we conducted a systematic literature analysis to determine the potential AEs, their frequency and the diversity specific to diet. Unlike previous reviews, this analysis was not limited to specific indications or age groups. Prior systematic reviews and meta-analyses (e.g. [2, 18–20]). primarily examined the use of KDs in pediatric epilepsy patients. In contrast, the present analysis considers various forms of KDs (CKD, MAD, LGIT, and MCT-KD), a broad age spectrum, and different medical applications. The aim was to provide nutritionists and medical professionals with an evidence-based foundation on which to evaluate the benefits and risks of using KDs.

## Materials and methods

### Literature search

The systematic literature search was conducted according to the PRISMA guidelines [21]. The search was performed in the electronic database PubMed and covered the period from November 2019 to November 2024. The complete search term and the filter options used are shown in Appendix table A1. The analysis aimed to document the range and prevalence of AEs associated with KDs, rather than evaluating causal relationships.

Literature was managed using Citavi Version 6. Intervention studies in humans of all ages, genders, and ethnicities (excluding pregnant women) were included if they administered a KD exclusively oral for at least 24 h. A KD was defined as a diet in which fat constitutes ≥ 60 energy percentage (E%) of total calories or has a fat: carbohydrate+protein ratio of ≥ 1.5:1 and carbohydrates constitute ≤ 10 E% of total calories or ≤ 60 g per day. Studies without information on AEs or without a detailed description of the symptoms or macronutrient intake were excluded. In vitro and animal studies, observational studies, reviews, meta-analyses, posters, and unpublished or incomplete studies were also excluded (see inclusion and exclusion criteria in Table 1).

**Table 1:** Inclusion and exclusion criteria of the systematic literature analysis

Inclusion criteria	Exclusion criteria
All individuals, regardless of age or underlying disease (except pregnant women)	In vitro and animal studies, pregnant women
Ketogenic diet (fat ≥ 60% of energy intake or ≥ 1.5:1 ratio; carbohydrates ≤ 10% of energy intake or ≤ 60 g/day)	Fat < 60 E% or < 1.5:1 ratio or carbohydrates > 10 E% or > 60 g/day)
Duration ≥ 24 h, exclusively oral food intake	< 24 h intervention, enteral or parenteral nutrition
Completed and published intervention studies	Unpublished or incomplete studies, observational studies, reviews, meta-analyses, posters
Survey and presentation of adverse events that occurred	No information on adverse events

### Data extraction

Data extraction was performed in Microsoft Excel (version 2108). The following data were recorded: authors and year of publication, study type, number of participants, age, gender, body mass index (BMI), underlying disease, type of diet, daily carbohydrate and fat intake, use of ketosis-promoting substances (e.g. MCT, exogenous ketone bodies), concomitant pharmacotherapy, and duration of intervention. Dropout rate and participants lost to follow-up were also recorded. Mean values and standard deviations were extracted from studies. If mean values and standard deviations were not specified, the median or range was recorded instead. Disease-specific and general hematology laboratory values were not considered.

### Data analysis

The reported AEs were summarized and evaluated according to the Common Terminology Criteria for Adverse Events (CTCAE) version 5.0 [22, 23]. Irrelevant CTCAE classes (e.g., hematology and endocrinology) were excluded. To allow for direct comparison changes in body weight, weight loss and weight gain were presented as separate categories, deviating from the CTCAE system. Fourteen medical disorder categories were identified based on the evaluation: gastrointestinal; musculoskeletal; psychiatric; neurological; dermatological; respiratory; infectious; weight gain; weight loss; metabolic and nutritional; general; cardiovascular; renal; and reproductive (see Appendix B).

For the analysis according to KD type five groups were formed: classic KD (CKD), KD (KD), modified Atkins diet (MAD), medium-chain triglyceride (MCT)-based KD (MCT-KD), and low glycemic index treatment (LGIT). Classification was made based on the designation by the authors of the individual studies. For the purposes of the evaluation, it should be noted that all KDs were classified as MCT-KDs, which used a ketosis-promoting substance.

The descriptive evaluation was performed in Microsoft Excel, including the calculation of the mean, median, standard deviation, and range. Due to a lack of available data, the hybrid mean (the arithmetic mean of the mean and median) was used for BMI and population age. Any AEs that were only reported in connection with study discontinuation were recorded descriptively and excluded from the calculation of the mean number of AEs per person (AE total/N total). No separate subgroup analysis was performed. Events classified by the authors as being unrelated to the intervention were marked as such. Only normocaloric interventions were considered when calculating the mean weight change.

## Results

### Search results

**Fig. 1:**
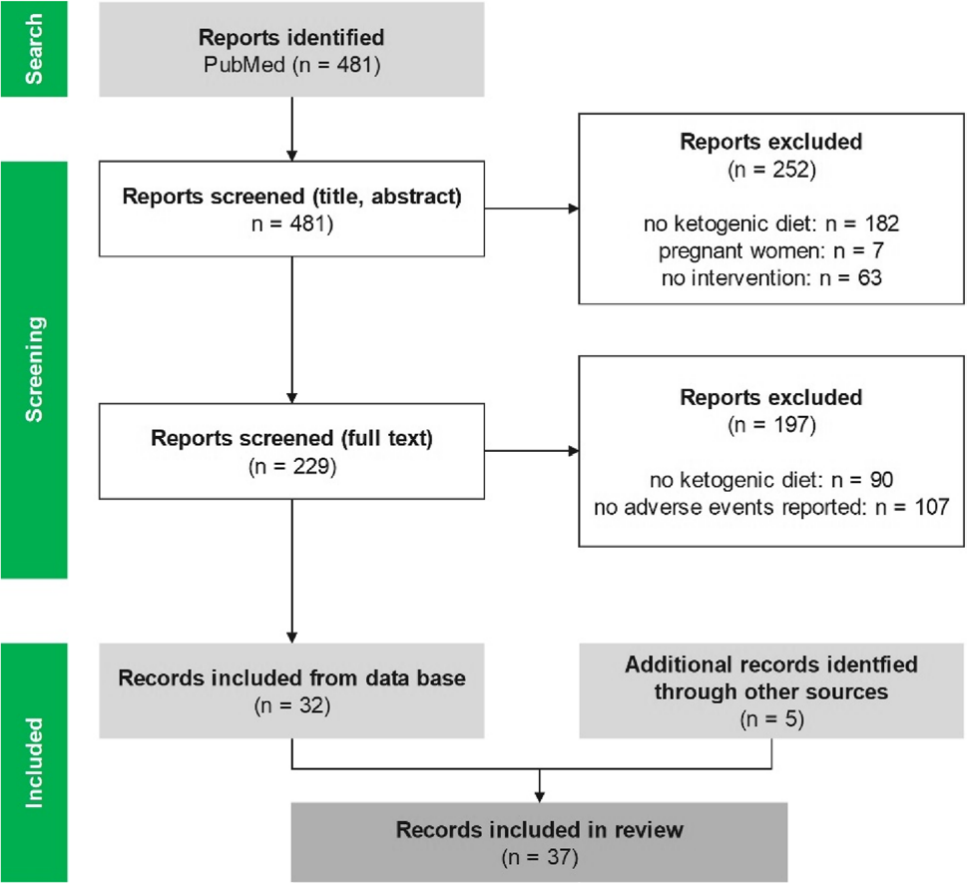
Flow chart of the literature search and screening in accordance to the PRISMA 2020 flow diagram for systematic reviews (modified according to [21])

A total of 481 articles were identified. Following title-abstract screening, 252 articles were excluded. These were excluded due to a non-ketogenic dietary intervention (*n* = 182), the presence of pregnant women (*n* = 7) or a study design without an intervention (*n* = 63). A subsequent full-text analysis of the remaining 229 articles resulted in an additional 197 articles being excluded because they either did not include a ketogenic dietary intervention (*n* = 90) or did not provide information on AEs (*n* = 107). Finally, 32 articles from the database search and five additional articles from other sources were included (see Fig. 1). This equates to 37 articles and 36 studies, since the study by Khodabakhshi et al. was published twice.

### Study characteristics

In accordance with the inclusion criteria, all of the included studies were prospective intervention studies published between 2019 and 2024. The studies originated from 14 countries. Of these, 23 were randomized controlled trials, six were non-randomized controlled trials, and seven were uncontrolled trials.

The 36 studies resulted in 42 ketogenic interventions, each lasting between 0.4 and 104.4 weeks (19.45 ± 24.01 weeks). The sample was distributed as follows: KD (*n* = 20; 47.6%), MAD (*n* = 9; 21.4%), MCT-KD (*n* = 7; 16.7%), CKD (*n* = 4; 9.5%), and LGIT (*n* = 2; 4.8%).

The average daily carbohydrate intake was 29.66 ± 16.21 g (7.5–50 g; 7.78 ± 2.14 E%) and the average daily fat intake was 72.24 ± 6.5 E% (60–80 E%). The highest carbohydrate value was found in the LGIT at 50 g per day, and the lowest in the CKD at 7.5 g per day.

In addition to the seven intervention groups (*N* = 42), in which the study population was defined as healthy, the interventions were based on the following underlying diseases (*n* = 35): epilepsy (*n* = 15, 42.86%), malignancies (*n* = 9, 25.71%), Parkinson’s disease (*n* = 3, 8.57%), glycogen storage disease type V (GSD type V; *n* = 2, 5.71%), bipolar disorder (*n* = 2, 5.71%), and one case each of multiple sclerosis (2.86%), obesity (2.86%), type II diabetes mellitus (2.86%), and liver disease (nonalcoholic fatty liver disease [NAFLD], 2.86%). The number of participants ranged from six to 80 (27.67 ± 18.96) and participant ages ranged from 1.23 to 67.3 years (32.63 ± 22.4 years). The mean BMI was 26.74 ± 3.57 kg/m² (21.1–34.0 kg/m²). The study population was predominantly underage in the CKD, LGIT, and MAD studies. On average, the gender distribution was 51.18 ± 26.96% male, 48.7 ± 26.89% female, and 0.12 ± 0.75% non-binary. The latter category was represented only in the study by Sethi et al. [24].

### Ketone body measurement

Ketone bodies were measured in the blood or urine of participants in 20 of the 42 interventions (47.62%). The metabolite measured and the time of measurement varied. While most studies determined BHB in serum, two studies measured AcAc in serum [25, 26] and three other studies reported unspecified “ketones” without specifying the substances [27–29]. As there were no uniformly comparable values available for AcAc, a quantitative evaluation was not possible. Serum BHB concentrations ranged from 0.19 mmol/L [30] to 2.98 mmol/L [31] (median = 0.91 mmol/L). Eight studies found BHB or total ketone concentrations of at least 1.0 mmol/L [25, 26, 31–34]. Of these, two studies, one MCT-KD and one CKD, measured mean values above 2.0 mmol/L [26, 31]. Due to the heterogeneous measurement times and methods, it was not possible to calculate a total mean value with a standard deviation.

### Adverse events, dropout, and number of adverse events

A total of 51 different AEs were reported in the included studies. Regardless of diet type or underlying disease, the dropout rate across all studies was 17 ± 15% (median 19%; 0–67%). CKD had the highest dropout rate (22 ± 18%), while LGIT had the lowest (5 ± 6%). The percentage of participants with at least one documented AE was 43 ± 30% (median 37%; 0–89%). The MAD group had the highest frequency of at least one AE (55 ± 43%), while the LGIT group had the lowest frequency (21 ± 8%). On average, participants experienced 0.91 ± 0.71 AEs (median 0.84; 0–3.3), and this number varied considerably between the different diets (LGIT: 0.25 ± 0.14; MCT-KD: 1.4 ± 0.72). The highest dropout rate among underlying diseases was observed in malignant patients on a KD (35 ± 22%), whereas no dropouts were recorded in healthy participants on an MCT-KD or patients with Parkinson’s on a KD. The proportion of participants with at least one AE was highest for GSD type V patients on a KD (83%) and lowest for epilepsy patients on a KD (14%). The highest AE rate per person was found in participants on an MCT-KD (1.4), and the lowest rate was found in participants on a LGIT (0.25) (see the key findings in Table 2).

**Table 2:** Dropout rate, proportion of participants with ≥ 1 adverse event, and mean number of adverse events per person associated with the most common adverse events stratified by diet type

Diet type	Dropout (%)	Proportion with ≥ 1 AEs* (%)	AEs* per person (Ø)	Most common AE*-category
CKD	22 ± 18	38	0.83	neurological
KD	17 ± 13	45	0.99	gastrointestinal
LGIT	5 ± 6	21	0.25	metabolic/nutritional
MAD	19 ± 20	55	1.11	gastrointestinal
MCT-KD	15 ± 12	49	1.40	gastrointestinal

*AE = adverse event

**Fig. 2:**
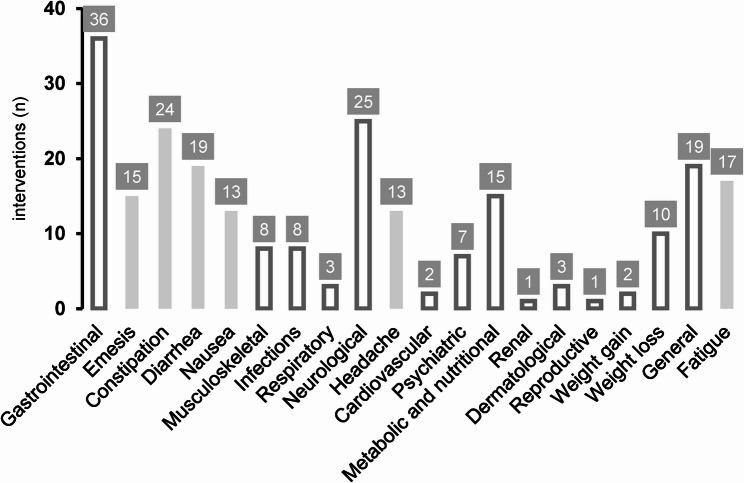
Number of interventions with adverse events, categorised by medical disorder. The main categories, based on CTCAE, are shown in white with dark gray borders. Individual adverse events are shown as light gray subcategories. Only those reported in > 10 interventions are displayed

According to the CTCAE categories, gastrointestinal AEs were reported in 36 out of 42 (86%) ketogenic interventions, with constipation (57%), diarrhea (45%), emesis (36%), and nausea (31%) being the most common. Neurological disorders occurred in 60% of interventions, followed by general symptoms (45%), metabolic and nutritional problems (36%), infections (19%), and musculoskeletal symptoms (19%). The rare categories (occuring ≤ 5% of cases) included cardiovascular, renal, and reproductive AEs (see Fig. 2).

Due to missing data, the evaluation of total AEs could only include 32 out of 42 interventions. A total of 743 AEs were recorded. The largest group was gastrointestinal symptoms with 297 cases (40%). Constipation was the most frequently reported individual AE within this category, accounting for 23.9% of all recorded AEs. This was followed by neurological disorders (*n* = 126; 17%), metabolic and nutritional problems (*n* = 87; 11%), infections (*n* = 74; 10%), and general symptoms (*n* = 62; 8%). Less common were respiratory symptoms (*n* = 24; 3%), weight loss (*n* = 17; 2%), psychiatric symptoms (*n* = 17; 2%), reproductive disorders (*n* = 13; 2%), and musculoskeletal AEs (*n* = 7; 1%). Fewer than 1% of total AEs were attributable to weight gain (*n* = 6; 0.8%), dermatological symptoms (*n* = 5; 0.7%), cardiovascular disorders (*n* = 3; 0.4%), or renal disorders (*n* = 2; 0.3%).

**Fig. 3:**
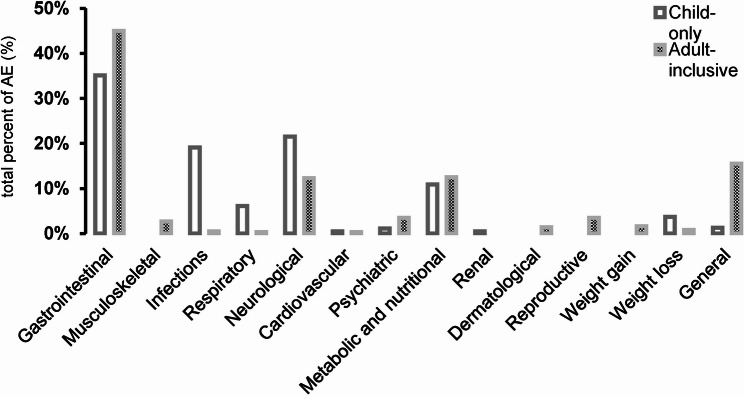
Distribution of total adverse events by CTCAE category, stratified by study age group. ‘Child-only’ refers to studies enrolling exclusively < 18 years, while ‘Adult-inclusive’ refers to studies enrolling adults (adult-only or mixed-age). Stratification is at the study level (not the individual level). Only interventions with complete AE counts were included (32/42)

Nine studies were conducted exclusively with participants under the age of 18 [31, 35–42]. The remaining 17 studies included a population of various ages, including adults. Gastrointestinal AEs accounted for 35% of all recorded AEs in children and 45% in adults. Musculoskeletal symptoms were only reported in studies with an adult population (3%). Infections occurred significantly more frequently in children (19%) than in adults (0.5%). Respiratory symptoms accounted for 6% of all AEs in children and 0.3% in adults. Neurological AEs occurred in 22% of children and 12% of adults. Cardiovascular AEs occurred in 0.5% of children and 0.3% of adults. Psychiatric symptoms accounted for 1% of total AEs in children and 4% in adults. Metabolic and nutritional AEs accounted for 11% of total AEs in children and 13% in adults. Renal AEs only occurred in children (0.5%). Conversely, reproductive (4%), weight gain (2%), and dermatological (1%) AEs only occurred in adults. Weight loss accounted for 4% of total AEs in children and 0.8% in adults. 16% of adults and 1% of children reported general symptoms (see Fig. 3).

### Adverse events by diet type

A total of 281 AEs were recorded from nine MAD studies, 198 from 13 KD studies, 197 from four CKD studies, 41 from four MCT-KD studies, and 26 from two LGIT studies. The most common AEs in CKD were infections (*n* = 65; 33%), followed by neurological (*n* = 57; 29%), gastrointestinal (*n* = 35; 18%), respiratory (*n* = 23; 12%), and metabolic and nutritional (*n* = 11; 6%) AEs (see Appendix C Table A2). Cardiovascular, renal, and general AEs each occurred in 1% of cases (*n* = 2). Among KD participants, gastrointestinal AEs accounted for the highest proportion (*n* = 75; 38%). These were followed by general symptoms (*n* = 42; 21%), neurological symptoms (*n* = 32; 16%), metabolic and nutritional symptoms (*n* = 29; 15%), and psychiatric symptoms (*n* = 10; 5%). The lowest incidence was observed for musculoskeletal AEs (*n* = 4; 2%), dermatological AEs (*n* = 2; 1%), weight loss (*n* = 2; 1%), cardiovascular symptoms (*n* = 1; 0.5%), and weight gain (*n* = 1; 0.5%).

The most common AEs associated with LGIT were metabolic and nutritional symptoms (*n* = 8; 31%) and gastrointestinal symptoms (*n* = 7; 27%). Infections, weight loss, and general AEs occurred at an equal rate (each: *n* = 3; 12%), followed by neurological symptoms (*n* = 2; 8%). Gastrointestinal AEs accounted for the largest proportion of MAD (*n* = 162; 58%). Metabolic and nutritional symptoms (*n* = 33; 12%) and neurological symptoms (*n* = 32; 11%) occurred significantly less frequently. The following AEs also occurred, in descending order: reproductive (*n* = 13; 5%), weight loss (*n* = 12; 4%), general (*n* = 8; 3%), psychiatric (*n* = 7; 3%), infections (*n* = 5; 2%), weight gain (*n* = 5; 2%), dermatological (*n* = 3; 1%), and musculoskeletal (*n* = 1; 0.4%). In MCT-KD, gastrointestinal AEs were also the most common (*n* = 18; 44%). Other common AEs included general (*n* = 7; 17%), metabolic and nutritional (*n* = 6; 15%), musculoskeletal (*n* = 5; 12%), and neurological (*n* = 3; 7%) symptoms. Infectious and respiratory AEs were the least prevalent (each: *n* = 1; 2%).

### Duration of adverse events

Only seven of the 36 studies (19%) specified the duration of AEs (see Table 3) [24, 37, 43–47]. The durations ranged from one week (LGIT) to six weeks (CKD), with an average of 3.5 ± 1.93 weeks (median 3.0; 1–6).Two studies differentiated the duration of individual symptoms: Needham et al. reported constipation lasting one week, fatigue lasting over two weeks, and diarrhea lasting over half a week [46]. Sethi et al. described constipation lasting over two weeks, fatigue lasting an average of 1.86 weeks, and headaches lasting one week [24]. Gastrointestinal and general AEs were most often characterized as “temporary”. General symptoms such as fatigue and headaches, frequently occurred during the keto-adaptation phase, i.e., the first two to seven days, and were summarized in some studies as “keto flu” [8, 10, 15].

**Table 3:** Overview of the duration of reported adverse events in the included studies on ketogenic diets (*n* = 7)

Study (author, year)	Type of diet	Duration of adverse events (weeks)
Augustus et al. [43]	KD	6
Brenton et al. [44]	MAD	2
Harvey et al. [45]	KD	2.57
Lakshminarayanan et al. [37]	LGIT	1
Needham et al. [46]	KD	Constipation: 1; Fatigue: 2; Diarrhea: 0.5
Sethi et al. [24]	KD	Constipation: 2; Fatigue: 1.86; Headache 1
Souza Neves et al. [47]	MAD	4

### Change of body weight

Data on changes in body weight were extracted from 21 of the 42 interventions (50%) involving adult study populations. For a further ten interventions, weight loss was described qualitatively but not quantified [18, 31, 35, 38, 41, 42, 48, 49]. Eleven interventions (26.2%) had no available information on weight development. Two studies involving hypocaloric diets were excluded from the calculation as a direct influence on weight was expected due to reduced energy intake [28, 50]. One study involved a diet with two days of fasting per week [50], and the other involved an intervention explicitly described as a “calorie-restricted diet” [28]. Across all normocaloric interventions evaluated, there was a tendency towards weight loss. The average weight loss was 4.56 ± 2.65 kg (median 3.9 kg; 0.1 [27] – 10 kg [24]). The mean intervention duration was 6.08 weeks. Converted to weekly averages, this equated to an average weight loss of 0.75 ± 0.73 kg (median 0.57 kg; 0 [51] – 2.86 kg/week [26]). Due to the limited and heterogeneous data available, a direct correlation between diet type and extent of weight loss could not be determined. Weight gain was reported as an AE in only two studies (both adult cohorts), with the underlying conditions being multiple sclerosis (MS) [44] and non-alcoholic fatty liver disease (NAFLD) [52], respectively.

## Discussion

Failure to adhere to the maximum allowable carbohydrate intake in KDs results in lower, or otherwise implausible, ketone body concentrations. However, many studies on KDs show deficiencies in the measurement of ketone bodies or lack data [51, 53]. The included study by Sethi et al. classified participants as adherent or non-adherent. Of the 21 participants, 14 (66.7%) were classified as adherent, meaning that at least 80% of their measured ketone bodies were above 0.5 mmol/L [24]. Participants in the McCullough et al. study were the only ones whose serum values were below 0.5 mmol/L [30]. This can be explained by their relatively mild KD compared to those in other included studies. Additionally, given the standard deviations in carbohydrate consumption, not all subjects achieved the target value (week 4: 50 ± 18 g; week 8: 45 ± 20 g); and measured fat intake was only 60 E% after eight weeks. Only nine interventions showed blood ketone levels above 1.0 mmol/L, which may indicate insufficient dietary adherence among the study populations. Some participants in ketogenic interventions do not achieve diet-induced ketosis, and insufficient compliance is a fundamental problem in this field of research [11]. Furthermore, fewer than 50% of the included studies measured and published ketone bodies in blood or urine, representing a substantial qualitative limitation that hinders the evaluation of study results [53].

Due to their long-term application and challenging implementation, dietary interventions often have a high rate of non-compliance [54]. KDs are often described as poorly tolerated, highly complex, restrictive, and associated with AEs, intolerances, and ineffectiveness, resulting in low compliance [3, 19, 55]. One objective factor for assessing compliance is the dropout rate. In a scoping meta-review of the use of KDs in children with epilepsy, Abbasi et al. found that 45.7% of participants were still following the diet after one year. This percentage decreased to 27–29.2% after two years. By contrast, the dropout rate was approximately 16% with comparatively short intervention durations of three months [55]. Additionally, a meta-analysis of pediatric epilepsy revealed that dropout rates for CKD and MCT-KD (10–26%) were significantly higher than for the milder variant, MAD (2–14%). In addition to the aforementioned reasons for discontinuation, unintentional weight loss was also cited [19]. Other sources report dropout rates of up to 50% for adults on a very restrictive CKD and 28–42% on a MAD [56, 57]. CKD is particularly associated with low compliance and a high rate of treatment discontinuation in adults [14]. As previously mentioned, the occurrence of AEs, especially gastrointestinal symptoms, can lead to discontinuation of therapy [20, 58]. However, it should be noted that AEs resulting from other therapies (e.g., chemotherapy or radiation therapy for malignant tumors), or the disease itself cannot necessarily be attributed to the KD as the cause [59]. The dropout rates shown for short-term KD use correspond to the average dropout rate of 17% determined in this study. There is also a tendency towards an increased rate of dropouts with stricter diet variants. Due to the limited number of studies, it is difficult to draw conclusions about the influence of individual underlying diseases on the dropout rate. However, the highest percentage of dropouts was found among patients with malignant tumors on a KD, which may be related to the severity of the underlying disease. The cancers included covered different stages and tumor types, such as breast, prostate, colon/rectum, cervical, lung, brain, and ovarian cancers, which increases the heterogeneity of the results further [18, 28, 43, 50, 60–64].

During the study selection process, it was noted that there was a lack of information on AEs. This does not necessarily mean that the interventions were free of AEs. Two systematic reviews on epilepsy and the KD found that all included studies reported AEs [2, 19]. Therefore, it can be concluded that diet-related AEs are underreported to a high degree. This finding is consistent with methodological analyses showing that fewer than half of all KD interventions systematically searched for or classified AEs according to standardized criteria, such as CTCAE [53]. Based on the present analysis, the probability of experiencing at least one AE while on a KD was 43% (0–89%). In addition to general underreporting, this limited presentation of AEs can be explained by the fact that some studies only documented serious AEs [39], and others only reported them in relation to dropout data [32]. A systematic review of KD in mitochondrial diseases revealed higher rates of symptom-independent or non-specific AEs. AEs were observed in 65% of individuals with mitochondrial diseases, which is comparable to the incidence of AEs in individuals with PDH deficiency (68%) on a KD [58]. In a retrospective analysis, 80% of children with epilepsy experienced AEs. It was noted that younger children are at greater risk [65]. Among the various underlying diseases, patients with GSD type V were found to have the highest tendency (83%) to experience at least one AE, while those with epilepsy had the lowest (14%). However, this data comes from only one study, so its significance should be considered limited. Patients with malignant diseases on a MCT-KD experienced the highest number of AEs per participant (Ø 2.4), while epilepsy patients on an LGIT experienced the lowest number (Ø 0.25). Overall, LGIT showed a low tendency for AEs, which can be explained by its comparatively low dietary strictness [37, 38]. The high value for malignant patients on MCT-KD is based on the description by Martin McGill et al. However, it should be noted that the patient group was undergoing oncological therapy, which is likely to result in further AEs from chemotherapy or radiation therapy that could act as confounders [18].

Overall, the diet strictness scores corroborate the well-established correlation between dietary restriction, tolerability, and symptom severity [4, 55, 66]. Analysis of AEs revealed that the MCT-KD had the highest AE value of 1.4 ± 0.72 per person. One possible reason for this is the high dose of MCT used, as it has repeatedly been associated with an increased incidence of gastrointestinal symptoms such as diarrhea, nausea, and abdominal cramps, in the literature [67, 68]. CKD also showed a comparatively high number of AEs. However, it should be noted that CKD had the longest average intervention period (approximately 59 weeks), so an increased cumulative probability of AEs cannot be ruled out with longer study durations. Additionally, the detailed and systematic recording of AEs in individual studies, particularly in Schoeler et al., may have led to an overestimation of the average number of AEs, as minor and clinically less relevant symptoms were also recorded [31].

The variety of observed AEs can be explained by the physiological changes that occur during KD, which affect nearly the entire human body [69, 70]. These changes include alterations in energy metabolism, hormonal shifts, electrolyte imbalances, and gastrointestinal and neurological reactions, which may manifest either acutely or in the long term. A systematic review of KDs for childhood epilepsy identified over 40 associated symptoms, highlighting this variability [20]. The identification of 51 AEs in the present analysis is primarily due to the inclusion of different clinical presentations and age groups. This illustrates that the range of possible AEs is not limited to specific populations. This provides a broader basis for risk assessment and the development of preventive and therapeutic measures to minimize undesirable effects when implementing KDs. Based on the available data short-, medium- and long-term AEs that were already known and mentioned in the guidelines could be confirmed [8, 10, 14, 16]. However, it was not possible to determine the occurrence of pancreatitis, optic neuropathy, QT interval prolongation, osteopenia or hepatitis. This is likely due to the short intervention periods of the included studies (∅ 19.45 weeks). Notable effects on bone mineralization and growth, for example, can only be expected after 12 months [66]. Furthermore, the lack of consistent reporting of certain assessments required to detect AEs, such as bone mineral density testing and echocardiography, limited comparability and potentially resulted in an underestimation of long-term events. Additionally, AEs not identified in this analysis were sometimes described as “very rare” [14]. With a total of 1,162 participants, the present sample size may have been too small. Previous reviews and meta-analyses did not describe symptoms such as depression, anxiety, neurological seizures, dystonia, acne, eczema, back pain, respiratory AEs, anorexia, amenorrhea, and dysmenorrhea. Some of these symptoms may be absent from the current literature because they are not diet-related. The authors of the studies stated a non-dietary relationship with pulmonary embolisms, eczema, infections, seizures and back pain [18, 25, 50, 71]. In addition to the effects of the underlying disease, medication may also be a relevant factor. One example is the anticonvulsant valproic acid, which impairs fatty acid oxidation and interacts with KDs [20]. However, since the use of medications and concomitant therapies was reported inconsistently, it was not possible to perform a quantitative subgroup analysis by medication class in this review.

Due to the limited data available, it was not possible to categorize the symptoms by time period. In particular, the absence of standardized surveys at the beginning, during and end of the intervention makes it difficult to clearly assign symptoms to specific time periods. However, the analyzed data indicate that short-term AEs last an average of three and a half weeks. Gastrointestinal and general AEs, in particular, were increasingly described as “temporary” [24, 46]. General symptoms such as fatigue and headaches are consistent with “keto flu”, a group of symptoms associated with metabolic adaptation in the early stages of a KD. “Keto flu” occurs within the first two to seven days [8, 10, 15]. A systematic review of the impact of KD on quality of life in chronic diseases and individual differences in the keto induction phase also supports this conclusion [1].

A total of 86% of the included studies reported gastrointestinal AEs, accounting for 40% of all recorded AEs. Desli et al. found that all included studies reported gastrointestinal AEs, which differs slightly from the results of the present analysis [3]. Several publications confirm both the number of AEs identified and their proportion of the total number of AEs [2, 12, 19, 20, 55, 65]. A review of the use of KDs in children revealed that up to 50% of participants experienced gastrointestinal AEs [12]. Lin et al. retrospectively reported a slightly lower incidence of 42%, and Sourbron et al. determined an incidence of 30% in a systematic review of children [19, 65]. These results are consistent with those of the present analysis. Directly comparing the number of AEs is complicated by the heterogeneous nature of the studies. In addition to pediatric data, the Cochrane review by Martin-McGill et al., which included patients of all ages, consistently lists constipation, vomiting, and diarrhea as the most common AEs across KD variants [72]. Taken together with pediatric reviews, these results suggest that gastrointestinal AEs are the most frequently reported, regardless of age group or KD variant [4, 19, 20].

Constipation was the most prevalent single AE in the present analysis, accounting for 23.9% of the total number of AEs. This aligns with the results of several systematic reviews, in both children with epilepsy and adults on a KD [2, 4, 19, 20, 72]. Martin-McGill et al. reported constipation rates ranging from 15 to 46% among children on a KD [72]. The present findings are consistent with those reported by Wells et al., who also identified constipation as the most frequently reported AE in different groups following various dietary regimens [4]. Sourbron et al. and Martin et al. reported constipation rates of 15–46% and 20–46% for MAD, respectively [2, 19]. It is important to note that these reviews are partly based on the same studies, which explains the striking similarity in their findings. In comparison, the constipation rate in the review by Cai et al. is lower, at 13.2%. This discrepancy can be attributed to the fact that Cai et al. exclusively included CKD interventions and pediatric patients with refractory epilepsy [20]. A comparison of age groups revealed that children were less likely to experience gastrointestinal AEs than adults. This observation provides additional support for the disparities in outcomes reported by Cai et al. [20]. However, etiology remains unclear and requires further research.

Several AEs have been documented in relation to KDs and some studies have suggested a correlation with vitamin and mineral deficiencies. These imbalances may be exacerbated further by restrictive dietary choices and the restriction of carbohydrates [4, 9, 12, 66]. It is important to note that vitamin D, calcium, magnesium, and selenium, along with other trace elements, play a pivotal role. However, it has been observed that ensuring adequate vitamin D levels cannot be accomplished solely by adhering to a ketogenic or conventional diet [14, 16]. Consequently, current guidelines and reviews advocate the targeted supplementation of critical micronutrients at the start of a KD, particularly in children but increasingly in adults as well [9, 12, 66]. According to existing literature, regular laboratory diagnostic monitoring is essential for the early detection of potential deficiencies and can minimize the risk of corresponding AEs [12, 14, 66].

The present analysis reveals a lack of data on the incidence of severe AEs and dysmenorrhea. This may be due to the brief intervention periods, insufficient documentation of AEs and incorporation of gender-non-specific information into the primary studies. Utilising disparate study designs and populations, alongside occasionally discrepant information regarding AEs, constitutes a salient limitation in data analysis. In subsequent research concerning KDs, irrespective of the underlying indication, meticulous and standardized documentation of AEs is imperative to enhance comparability and transparency. The various dietary forms should be critically evaluated, as the included studies often lack detailed information on energy intake and macronutrients, which complicates comparative analysis further. Future studies should expand their search to include additional scientific databases, such as Web of Science or ScienceDirect, as the current approach focused exclusively on studies published on the database PubMed. Due to the heterogeneity of the studies and available data, a meta-analytic evaluation was not possible.

## Conclusion

The AEs of KDs are multifaceted and can vary significantly in terms of frequency. This discrepancy can be attributed to various factors, including differences between patients and the specific medical field of application. However, a significant contributing factor is the inconsistent and sometimes inadequate documentation of AEs in the included studies. Preliminary analysis suggests that there is a high probability of experiencing gastrointestinal, neurological and metabolic AEs. However, the symptoms seem to be mild and reversible. Severe AEs appear to be infrequent in general with short-term use and less intense forms of diet, such as MAD or LGIT. The results also indicate a correlation between the frequency and spectrum of AEs and the restrictiveness of the KD.

When prescribing and implementing KDs in clinical practice, it is imperative to perform a comprehensive risk-benefit assessment and ensure that patients are thoroughly informed about the potential AEs and strategies for their prevention. Regular medical check-ups and targeted supplementation of critical micronutrients must be prioritised particularly in children, to avert potential complications.

Future studies should systematically record AEs as a central outcome over extended periods of time, taking into account gender- and age-related differences, and utilizing standardized classification systems for AEs.

## Data Availability

The data supporting the conclusions of this article will be made available by the authors upon request by the corresponding author (T.F.; tobias.fischer@fh-muenster.de).

## Appendix A

**Table 4:** Search strategy, including database, search term, and filter

Database	Search Term	Filter
PubMed	“ketogenic” [Title/Abstract] OR “modified Atkins” [Title/Abstract] OR “low glycemic index diet” [Title/Abstract] OR “low glycemic index treatment” [Title/Abstract] OR “low carb” [Title/Abstract] OR “low carbohydrate*” [Title/Abstract] OR “medium chain fatty acid*” [Title/Abstract] OR “medium chain triglyceride*” [Title/Abstract])	Clinical Study, Clinical Trial, Clinical Trial, Phase I, Clinical Trial, Phase II, Clinical Trial, Phase III, Clinical Trial, Phase IV, Controlled Clinical Trial, Randomized Controlled Trial, from 2019 to 2024, Humans, English

## Appendix B

Classification of adverse events according to disorder categories (modified according to [22]):

- Gastrointestinal: emesis, constipation, diarrhea, nausea, abdominal pain, dyspepsia, colitis, reflux, hypersalivation, bad breath, dry mouth.
- Musculoskeletal: muscle pain, muscle cramps, back pain, cold extremities.
- Psychiatric: insomnia, depression, anxiety, irritability.
- Neurological: dystonia, seizures, headaches, somnolence, lethargy, dizziness, presyncope.
- Dermatological: eczema, acne, hair loss.
- Respiratory: dyspnoea, pulmonary embolism.
- Infections.
- Weight gain.
- Weight loss.
- Metabolic and nutritional: hunger, lack of energy, loss of appetite, anorexia, ketoacidosis, electrolyte disturbances, hypoglycemia, hyperuricemia, dyslipidemia.
- General: Fever, malaise, fatigue.
- Cardiovascular: hypotension, cardiac arrest.
- Renal: kidney stones.
- Reproductive: amenorrhea, dysmenorrhea.

## Appendix C

**Table 5:** Types of diets by adverse events according to CTCAE-categories [22] in percent

	CKD	KD	LGIT	MAD	MCT-KD
Gastrointestinal	17.8	37.9	26.9	57.7	43.9
Musculoskeletal	0.0	2.0	0.0	0.4	12.2
Infections	33.0	0.0	11.5	1.8	2.4
Respiratory	11.7	0.0	0.0	0.0	2.4
Neurological	28.9	16.2	7.7	11.4	7.3
Cadiovascular	1.0	0.5	0.0	0.0	0.0
Psychiatric	0.0	5.1	0.0	2.5	0.0
Metabolic and nutritional	5.6	14.6	30.8	11.7	14.6
Renal	1.0	0.0	0.0	0.0	0.0
Dermatological	0.0	1.0	0.0	1.1	0.0
Reproductive	0.0	0.0	0.0	4.6	0.0
Weight Gain	0.0	0.5	0.0	1.8	0.0
Weight Loss	0.0	1.0	11.5	4.3	0.0
General	1.0	21.2	11.5	2.8	17.1
